# Single cell sequencing: a distinct new field

**DOI:** 10.1186/s40169-017-0139-4

**Published:** 2017-02-20

**Authors:** Jian Wang, Yuanlin Song

**Affiliations:** Department of Pulmonary Medicine, Zhongshan Hospital, Fudan University, Shanghai, 200030 China

## Abstract

Single cell sequencing (SCS) has become a new approach to study biological heterogeneity. The advancement in technologies for single cell isolation, amplification of genome/transcriptome and next-generation sequencing enables SCS to reveal the inherent properties of a single cell from the large scale of the genome, transcriptome or epigenome at high resolution. Recently, SCS has been widely applied in various clinical and research fields, such as cancer biology and oncology, immunology, microbiology, neurobiology and prenatal diagnosis. In this review, we will discuss the development of SCS methods and focus on the latest clinical and research applications of SCS.

## Introduction

The majority of experimental and clinical results from cell culture or tissues are based on the assumption that all of the cells in a culture or tissue are homogeneous. The thriving omics fields of study (genomics, proteomics, transcriptomics, etc.) analyze and mine biomarkers mainly based on the bulk of cells or tissue samples. However, this averaging of messages always misses the critical information because the heterogeneity of the samples is ignored, while the nature of biology is diverse. Heterogeneity is generally explained at three different levels in the biological universe: first, there is heterogeneity in different organisms; second, there is heterogeneity in different organs or tissues from an organism; third, cellular heterogeneity exists in the same organ or tissue. In fact, the concept of cellular heterogeneity was proposed as early as 1957 [1]. Each cell was considered as a unique unit with molecular coding across the DNA, RNA, and protein conversions [2]. Thus, it is necessary to conduct studies, especially omics studies, at the single cell level.

A single cell is the smallest structural and functional unit of an organism. The estimated number of single cells in the human body is 3.72  ×  10^13^ [3]. The size or weight of a cell varies from different tissue backgrounds. The major components of a cell include water, inorganic ions, small organic molecules, proteins, RNA and DNA. However, the minute numbers of copies of a gene (10–12 M) in a single cell are more than enough for conventional genomic analysis [2, 4]. In 2009, the first single cell whole transcriptome sequencing (WTA) protocol was applied to analyze transcriptome complexity in individual cells [5]. Subsequently, single cell whole genome sequencing (WGS) was created in 2011 [6], single cell whole exome sequencing (WES) was developed in 2012 [7, 8], and single cell epigenomic sequencing was developed in 2013 [9]. Currently, single cell sequencing (SCS) has been applied in various research and clinical fields, and the top five areas of SCS studies in order are cancer, embryonic development, microbiology, neurobiology and immunology, according to the reported statistics [10]. The number of SCS publications in these five areas has been increasing every year. Thus, this article will enable us to have a deep and broad view of SCS methods and to focus on the latest application of SCS in basic and clinical research.

## Single cell isolation methods

**Fig. 1:**
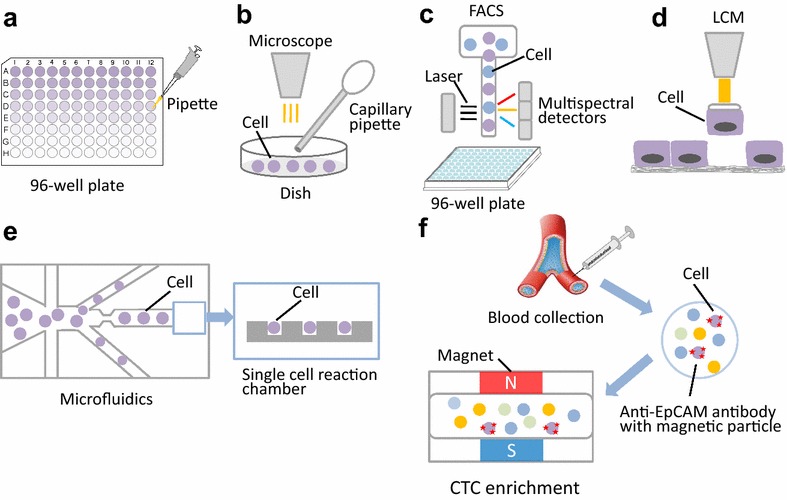
The current methods for single cell isolation. **a** Serial dilution. **b** Mechanical micromanipulation. **c** Laser capture microdissection (LCM). **d** Fluorescence activated cell sorting (FACS). **e** Microfluidics. **f** The representative platform for circulating tumor cells (CTCs) isolation: CellSearch

Isolating single cells from a tissue mass or from cell culture is the first key step prior to SCS. Currently, the alternative methods used to isolate single cells from abundant populations include serial dilution, mechanical micromanipulation, laser capture microdissection (LCM), fluorescence activated cell sorting (FACS), and microfluidics [11, 12]. Although serial dilution is the simplest method to obtain a single cell in a single well via serial double dilution, it is a coarse and imprecise method that is rarely used in SCS (Fig. 1a). Our team has tried to use this method to isolate a single cell from primary lung cancer cells in cell suspension and found that it was hard to control the quality and quantity [13].

Mechanical micromanipulation is a classic method to isolate uncultivated microorganisms or early embryos, and it involves using a capillary pipette to suck up a single cell from a cell suspension with visual inspection of cellular morphology and coloring characteristics under a microscope [13, 14] (Fig. 1b). The drawback of mechanical micromanipulation is that it is low-throughput and time-consuming and can cause cellular injury from mechanical shearing during manipulation [15]. Additionally, it often leads to a failure for an unskilled manipulator or misidentification of the cellular morphology under the microscope.

FACS is the most efficient and economical method to isolate hundreds of thousands of individual cells per minute based on their size, granularity and fluorescence properties [4] (Fig. 1c). The high-throughput, time-saving and automatic properties are the main advantage of FACS. Additionally, it allows researchers to isolate specific individual cells from heterogeneous cell samples by labeling the targeted cells with specific fluorescent antibodies [16], and it allows researchers to sort a single viral particle from a mixed viral assemblage for single viral genome sequencing [17]. BD Aria II/III (BD Biosciences, San Jose, CA, USA) and Beckman Coulter MO-FLO XDP cell sorter (Beckman Coulter, Brea, CA, USA) are two widely used commercial instruments for flow cytometry [11]. Our team has used the BD Aria III to sort individual living cells from lung cancer tissue single cell suspensions that were stained with carboxyfluorescein diacetate succinimidyl ester (CFSE) and sorted into 96- well plates [18]. However, a bulk population of the cells (at least 5 × 10^5^–1 × 10^6^/ml) should be prepared as sorting material, which is greatly limited in accommodating low-abundance cell subpopulations. The high-speed fluid and fluorescent dye can damage the viability of cells.

Microfluidics is a newly developed and highly integrated system that sequentially processes or manipulates small volumes of fluids (10^−9^–10^−18^ l) in channels with dimensions of tens to hundreds of micrometers to achieve single cell culture and sequencing, that has been applied to single cell experiments [19, 20] (Fig. 1e). Recently, various microfluidics platforms have emerged for single cell whole-genome, whole-transcriptome or epigenomics sequencing [21–23]. The advancement of microfluidics research has extended to separate nanoparticles, such as DNA isolation [24]. The advantages for microfluidics are the ability to input nanoliter-to-picoliter volumes of samples and to output accurate results with high resolution and sensitivity [19]. Additionally, microfluidics can provide parallel and timely analyses to make studies more efficient.

The main limitation of the above-mentioned methods is that the sample must be prepared in suspension and thus have lost the spatial location of the cells in the tissue. LCM overcomes this limitation and directly isolates single cells from tissue sections based on the cellular morphology (Fig. 1d). The targeted single cell can be stained with fluorescent or chromogenic antibodies for LCM [11]. The main drawbacks include low-throughput, slicing the cells during the course of tissue sectioning, and UV damage to nuclei from the laser [12].

The increasing number of studies on rare single cells (<1%) poses a challenge on the current methods for single cell isolation. Now, several new technologies have been developed to cover the shortcomings of the above-mentioned methods in rare single cancer cell isolation, such as CellSearch (Johnson & Johnson), MagSweeper (Illumina Inc.), DEP-Array (Silicon Biosciences), CellCelector (Automated Lab Solutions), and nanofabricated filters (CellSieve) [25]. The FDA-cleared CellSearch system is the most-advanced commercially available technology using anti-EpCAM ferrofluid and has been applied to the monitoring of patients with metastatic prostate, breast, or colorectal cancer in hospitals [26, 27] (Fig. 1f). MagSweeper is an automated immunomagnetic separation technology for enrichment of rare cells in mixed populations with high purity [28]. DEP-Array uses a microfluidics chip with dielectrophoretic cages to isolate single cells by charge [12, 29]. The CellCelector uses a robotic arm carrying a module to retrieve single cells from microwells for micromanipulation [30]. The CellSieve system can capture a variety of circulating tumor cells based on size discrimination instead of specific cell surface markers [31].

## Single cell sequencing methods

The advance in the next-generation sequencing (NGS) technologies has promoted the rapid development of SCS, including single cell whole-genome sequencing, single cell whole-exome sequencing, single cell whole-transcriptomic sequencing and single cell epigenomic sequencing [32–34].

### Single cell whole-genome/whole-exome sequencing

**Fig. 2:**
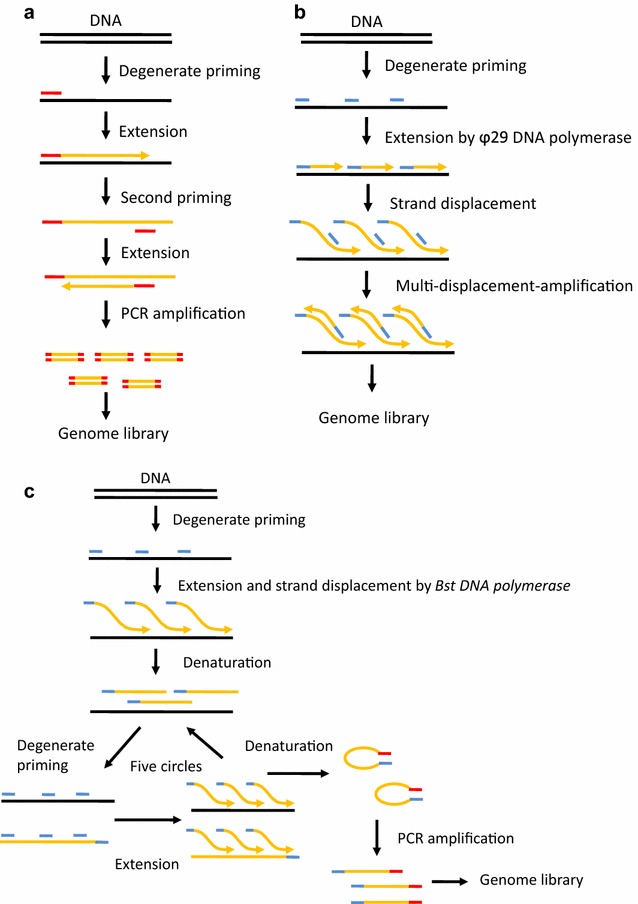
Schematic of the whole genome amplification methods for single cell sequencing. **a** Degenerate oligonucleotide-primed PCR (DOP-PCR). **b** Multiple displacement amplification (MDA). **c** Multiple annealing and looping based amplification cycles (MALBAC)

The amount of DNA (approximately 6 pg) in a single cell is insufficient to meet the demand for next-generation sequencing, and thus whole genome amplification (WGA) was developed to amplify the DNA by the hundreds of thousands [25, 35]. Recently, the alternative WGA technologies have polymerase chain reaction (PCR), multiple displacement amplification (MDA), or a combination of displacement pre-amplification and PCR amplification [36]. In PCR-based WGA methods, degenerate oligonucleotide-primed PCR (DOP-PCR) is the most widely used method to amplify the entire genome [37, 38]. The principle of DOP-PCR is to perform a low annealing degenerate primer extension on the DNA template and then to amplify the tagged sequences at a high annealing temperature [37, 39] (Fig. 2a). The main shortcoming for DOP-PCR is the low physical coverage (approximately 10%) of a single cell genome, which is prone to miss single-nucleotide polymorphisms (SNPs), but DOP-PCR is the optimal method for copy-number variations (CNVs) or aneuploidy detection because of the uniformity of amplification during WGS [12, 40, 41]. The established MDA technologies are based on the discovery of two specific DNA polymerases: Phi29 DNA polymerase isolated from the *Bacillus subtilis*, and Bst DNA polymerase isolated from *Bacillus stearothermophilus* [42–44]. The mechanism of MDA is to yield continuous strand displacement DNA amplification using Phi29 or Bst DNA polymerase and random primers under isothermal conditions [45] (Fig. 2b). Phi29 DNA polymerase has been considered the optimal choice for MDA because it shows higher efficiency, higher fidelity and a lower error rate compared with Bst DNA polymerase which has no proofreading activity [10, 46]. The advantages of MDA are that it has high single cell genome or exome coverage (>90%), which can accurately measure mutations at base-pair resolution and that it yields adequate quantities of product (average length >10 kb) from single cell genomic DNA in a short time with high fidelity [47]. However, the main drawbacks of MDA are uneven genome coverage, chimeric sequences, and contamination issues [15]. Multiple annealing and looping based amplification cycles (MALBAC) is the newly applied WGS method that combines quasi-linear strand displacement pre-amplification by a polymerase and exponential amplification by PCR [33] (Fig. 2c). Remarkably, MALBAC has low amplification bias and can achieve 93% genome coverage ≥1× and 25× mean sequencing depth in a single cell during WGS. Moreover, MALBAC shows higher efficiency to detect CNVs and SNPs for its improved uniformity and a lower allele dropout rate, compared with MDA [36]. The pitfall of MALBAC is the extremely high false positive rate for SNV detection because of the low fidelity of Bst DNA polymerase, and the loss of underamplified regions of the genome [48]. Another improved method, nuc-seq or single nucleus exome sequencing (SNES), has been developed to reduce the bias, this method combines flow-sorting of single G1/0 or G2/M nuclei, time-limited isothermal MDA, exome capture, and NGS [49, 50]. The main advantage of this method is the high detection efficiencies for single-nucleotide variations (SNVs) and indels benefiting from the high physical coverage (96%) of the single cell genome and exome [50].

### Single cell whole-transcriptomic sequencing

**Fig. 3:**
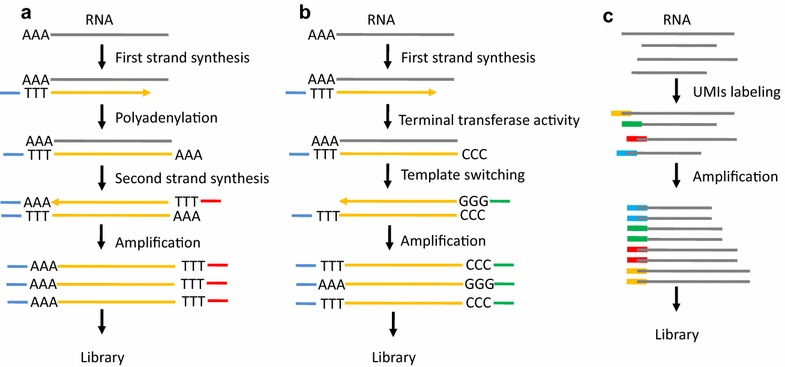
Schematic of the whole transcriptome amplification methods for single cell sequencing. **a** oligo dT-Anchor approach. **b** Template-switching approach (including SMART-seq, SMART-seq2 and STRT). **c** Unique molecular identifiers (UMIs)

It is estimated that the amount of total RNA or mRNA is only approximately 10 pg or approximately 0.1 pg, respectively, in a single cell [10]. Thus, WTA is a necessary step to construct a cDNA library for single cell transcriptomic sequencing. WTA has been applied to amplify RNA from a single cell to obtain the gene expression profile in microarray prior to the advent of NGS [51, 52]. Tang et al. [5] improved the single cell whole-transcriptome amplification method and used NGS instead of microarray to identify more genes and previously unknown splice junctions in single cells. The principle of this method is to use oligo dT primers conjugated to adapter sequences for reverse transcription and selective amplification of polyadenylated mRNA by PCR [5, 10, 53] (Fig. 3a). However, this method generates 3′-end skew bias during reverse-transcription to miss proximal splicing events [34]. Another WTA method, called SMART-seq, was developed to use Moloney murine leukemia virus (MMLV) reverse transcriptase to construct full-length cDNA libraries [54]. The two key features, template-switching and terminal transferase activity, of the enzyme can lead to adding a few non-templated C nucleotides to the cDNA and switching templates to transcribe the other strand [55] (Fig. 3b). The advantage of SMART-seq is to generate and amplify full-length cDNA from single cell RNA, leading to the detection of alternatively spliced exons [56]. The low sensitivity of SMART is the main shortcoming that was improved in a subsequently developed method, called SMART-seq2 [57]. Similarly, single cell tagged reverse-transcription (STRT) is based on the template-switching property of the reverse transcriptase to tag the 5′-end of cDNA [58]. This method enables researchers to compare gene expression profile differences without bias in multiple samples, but it yields a strong 5′-end bias. Cell expression by linear amplification and sequencing (Cel-seq) labels cDNA with a barcode and pools these cDNA from multiple single cells for in vitro transcription (IVT) to linearly amplify cDNA [59]. The CEL-Seq generates more reproducible, linear, and sensitive results in comparison with the PCR-based amplification method, but it yields a high 3′-end skew bias and loses the full spectrum of transcript variant detection [60]. Additionally, the unique molecular identifiers (UMIs) labeling technique is applied in single cell WTA to achieve quantitative single cell RNA sequencing [61] (Fig. 3c). This method obviously increases the accuracy in single cell whole-transcriptome sequencing by eliminating amplification bias. Recently, two new droplet-based RNA-seq technologies, named as Drop-seq and inDrop (indexing droplets), has been exploited to sequence in parallel thousands of single cells from a tissue [62, 63]. Each nanoliter-scale aqueous droplet is a tiny reaction chamber that contains a single cell, barcoded and UMI-labeled primers, and reaction buffer. STAMPs (Single-cell Transcriptomes Attached to Microparticles) is PCR amplified for sequencing in Drop-seq, while Cell-seq is used by inDrop for sequencing. The advantages of these methods are to differentiate the cell-of-origin of each mRNA which helps to develop single cell analysis in a complicated tissue, and the low technical noise that allows the analysis of thousands of different cells in parallel. The latest commercial platform—Chromium™ System from 10× Genomics—integrates the Gemcode platform, which separates long pieces of DNA into droplets to create barcoded sequencing libraries [64, 65]. The high efficiency and flexible throughput of this method allows researchers to dynamically detect transcriptional profiles of single cells at scale [66].

### Single cell epigenomic sequencing

Epigenomics is defined as a phenomenon that changes the final outcome of a chromosome without changing the underlying DNA sequence, including DNA methylation, histone modifications, chromatin packaging, small RNA, etc. [67]. Recently, single cell epigenomic sequencing studies are on the rise with the application of new single cell epigenomic sequencing methods. Single cell reduced representation bisulfite sequencing (scRRBS) integrates all the experimental steps before PCR amplification into a single-tube reaction to avoid unnecessary DNA loss and enables the detection of approximately 40% of the CpG sites in comparison with standard RRBS using thousands of cells [68, 69]. Another method, single cell bisulfite sequencing (scBS-seq), modifies the Post-Bisulfite Adaptor Tagging (PBAT) to perform bisulfite conversion prior to successive primer extension with oligo1 and oligo2 tagged random primers to generate amplicons [70]. The drawbacks of these methods are DNA loss, purification, and disability to discriminate 5mC from 5hmC for bisulfite conversion [34]. Moreover, single cell chromatin immunoprecipitation followed by sequencing (scChIP-seq) combines microfluidics, DNA barcoding and sequencing to collect low coverage maps of the chromatin state at single cell resolution [71]. Additionally, other methods have been developed for single cell epigenomic sequencing, such as Hi-C methods that characterize chromatin interactions in the genome of single cells [9], and single chromatin molecule analysis at the nanoscale (SCAN) that extracts single chromatin with fluorescent antibodies through fluidic channels [72].

## Application of single cell sequencing

### Cancer

Cancer heterogeneity comes from clone diversity and mutational evolution, which promote cancer cell survival and metastasis and confound the cancer diagnosis and treatment [10, 73]. A deep understanding of cancer heterogeneity can contribute to therapeutic decisions. Thus, SCS as an ideal tool has been increasingly applied to reveal intratumor heterogeneity in various primary tumors, such as breast cancer [6, 49, 74], lung cancer [75], brain cancer [76], colon cancer [33, 77], bladder cancer [78], acute myeloid leukemia [79, 80] and melanoma [81].

Navin et al. [6] first applied single nucleus sequencing (SNS) to study tumor population structure and evolution in two breast cancer cases by analysis of genome copy number variation. The results found punctuated clonal evolution in tumors and confirmed that metastatic cells emerged from a main advanced expansion. Another study used nuc-seq to find a difference in the pattern of occurrence for aneuploid rearrangements and point mutations in breast tumor evolution [49]. Furthermore, Eirew et al. [74] studied the dynamics of genomic clones in breast cancer patient xenografts at single cell resolution to indicate that genomic aberrations can be reproducible determinants of evolutionary trajectories. Interestingly, a single cell whole genome sequencing study for colon cancer identified an abundant amount of mutated gene SLC12A5 at the individual level, which was sparse at the bulk cells level, and discovered that colon cancer had a biclonal origin [77]. However, another study using single cell exome sequencing to reveal the evolutionary process in bladder cancer indicated that 66 individual bladder cancer cells were derived from a single ancestral cell, but they developed into two distinct tumor cell subpopulations with subsequent evolution [78]. Single cell exome sequencing was also applied to elucidate the intratumoral genetic characteristics at a single cell level in a kidney cancer [8]. Additionally, the clonal evolution has been studied in hematopoietic tumors. Hughes et al. [79]. sequenced single cells from three myelodysplastic syndrome (MDS)-derived secondary acute myeloid leukemias (sAMLs) to confirm the clonal architecture that was identified from the bulk sample analysis. Single cell exome sequencing revealed a monoclonal evolution in a JAK2-negative myeloproliferative neoplasm and further identified candidate gene mutations for neoplasm progression [7].

In addition to single cell DNA and exome sequencing applications, single cell RNA-seq has also been widely used to study clonal evolution in different cancers. Single cell RNA-seq demonstrated subclonal heterogeneity in xenograft tumor cells and found a candidate tumor cell subpopulation associated with anti-cancer drug resistance in lung adenocarcinoma (ADC) [75]. Equally, intertumor and intratumor heterogeneity was elucidated in melanoma by single cell RNA-seq [81]. Tirosh et al. [76] used single cell RNA-seq to find a new subpopulation marked with stem or progenitor cell-like characteristics, which supported developmental programs in oligodendroglioma. Furthermore, another study identified several rare tumor-related genes in squamous cell carcinoma of urinary bladder using single cell RNA-seq [81].

Recently, an array of studies that used SCS to understand the necessary knowledge of different rare circulating cancer cells have been published. Ni et al. [82] combined MALBAC with NGS to elucidate the CNV patterns for metastasis of cancer in circulating tumor cells (CTCs) from lung ADC. Lohr et al. [83] sequenced entire exomes of CTCs from two prostate cancer patients and observed 73% CTC mutations that were identified in bulk tissue. The results were consistent with another study that compared CTCs with tissue using WGS in prostate cancer [84]. Additionally, one study built a new system to assess the genomic heterogeneity of single CTCs from metastatic breast cancer patients and found a cell subpopulation related to drug resistance [85]. However, a recent study indicated that a targeted mutation detection rate is approximately 27.7% in CTCs from pancreatic cancer compared with bulk cells but is negative in white blood cells [86]. Furthermore, single CTCs studies based on whole RNA-seq have also been published. Lohr et al. [87] classified multiple myeloma (MM) and quantitatively assessed prognosis related genes using single CTC RNA-seq. Another CTC RNA-seq study revealed that noncanonical Wnt signaling took part in antiandrogen resistance in prostate cancer [88].

### Immunology

The heterogeneity of the immune system contributes to an efficient defense against a multitude of different pathogens [89]. The SCS technologies can help to define new classifications and differentiation trajectories of immune cells. CD4+ T helper cell, which play a key role in adaptive immune responses, are further investigated to unravel the heterogeneity of this celluar population at the single cell level. Mahata et al. [90] used single cell RNA-seq to reveal the extensive heterogeneity within the Th2 population and to identify a new Th2 cell subpopulation marked with Cyp11a1 that modulated the steroid synthesis pathway. Additionally, functional and structural studies of the T cell receptor repertoire have also benefited from SCS approaches. Dash et al. [91] developed a new method to sequence the TCRα and TCRβ chains from single CD8+ T cells. The data showed a characterized expression of TCRα for an influenza epitope. Another study combined TCRα and TCRβ sequencing with phenotypic analysis to reveal the clonal structure of T cells at the single cell level [92]. In addition to T cells, Shalek et al. [93] examined the mouse bone-marrow-derived dendritic cells (BMDCs), which is an important antigen-presenting cell subpopulation in the adaptive immune system, using single cell RNA-seq. The results indicated that hundreds of immune related genes displayed bimodal expression in single cells. Further study demonstrated that paracrine signaling from early-induced dendritic cells plays an important role in inflammatory program [94]. Although the application of SCS to study the immune system is limited at present, SCS has shown robust potential for defining immune cell subpopulations and for examining gene expression variability, differential splicing and gene-regulatory networks [89].

### Microbiology

The vast majority of microorganisms are uncultivated with current culturing methods which has extremely limited our ability to understand the biological diversity of the microbiome [95]. Recently, the difficulty in microbial research has been overcome with the development of SCS. The first study combined FACS with MDA to sequence single TM7 bacterial cells from the soil and gained a deep insight into the evolution and metabolism of these cells [96]. The member of TM7 phylum from the human mouth was also investigated in a similar method [97]. The subsequent study conducted the single cell genomic sequencing in other candidate uncultured phyla from different environments, including anoxic spring-derived OP11 [98], human microbiota-derived SR1 [99], hospital sink biofilm-derived TM6 [100] and hot spring sediments-derived OP9 [101]. In addition to sequencing the genome of various bacterial phyla, SCS can reveal the lifestyle and metabolism of uncultivated microorganisms, supporting the potential development of cultivation approaches and commercial applications. Marc et al. [102] sequenced over 70% of the genome of *Beggiatoa* from the surface of marine sediment and confirmed the chemolithoautotrophic physiology via investigating the pathway for sulfur oxidation, oxygen and nitrate respiration, and carbon metabolism. The findings supported the establishment of a particulate cultivating environment in which there was coexistence of different members of the microbial community and some missing supplementary materials [95]. In another study, Mason et al. [103] used MDA to sequence and assemble the single cell genome of *Oceanospirillales* from seawater after the Deepwater Horizon oil spill and identified enzymes that can degrade crude oil.

### Prenatal diagnosis

The application of SCS to prenatal diagnosis, including pre-implantation genetic diagnosis (PGD) and non-invasive prenatal diagnosis (NIPD) has greatly increased the opportunities for healthy birth [32]. Recently, SCS has been widely used to detect aneuploidy and SNPs in prenatal diagnosis. Well et al. [104] used a rapid WDA protocol to diagnosis of aneuploidy in embryo biopsy with high accuracy and cost-efficiency. In another study, Fiorentino et al. [105] confirmed the validation and accuracy of a single cell NGS-based method for aneuploidy screening in single blastomeres. In the subsequent study, they compared this protocol with array comparative genomic hybridization (array-CGH) and demonstrated that a single cell NGS-based method improved the aneuploidy detection with high-throughput, automation and reliability [106]. Furthermore, Vera-Rodríguez et al. [107] used single cell NGS to investigate the distribution patterns of segmental aneuploidies in trophectoderm biopsy. The efficiency of NGS in the detection of pure and mosaic segmental aneuploidies equated with that of CGH. Lu et al. [108] used MALBAC to sequence 99 sperm from an Asian male to detect aneuploidy and single nucleotide polymorphisms. The same method was used to accurately detect aneuploidy and SNPs in a single oocyte [109]. Additionally, using NIPD as a safe and reliable method to identify affected fetuses before birth is becoming increasingly popular for clinical and research applications in combination with NGS technologies. Zhang et al. [110] used low-coverage massively parallel sequencing to detect CNVs in four single cells from peripheral blood. The sensitivity and specificity for CNVs and aneuploidies were 99.63 and 97.71%, respectively. Hua et al. [111] used WGA and Illumina MiSeq to sequence single fetal nucleated red blood cells from placental villi and to diagnose aneuploidy in 5 cases in 10 single cells.

### Neurobiology

Defining neuronal heterogeneity is an enormous task in nervous research [112]. SCS has been increasingly used to understand neural cell diversity and to classify neurons. Many studies have been reported to use single cell RNA-seq to classify the type of neurons in various regions of the mouse nervous system, including monoaminergic systems, dorsal root ganglia, cortex and retina [112]. Okaty et al. [113] used single cell RNA-seq to distinguish serotonergic neurons from five hindbrain rhombomeres and confirmed the subpopulation grouped from the population-scale transcriptomes. Additionally, the subtype-specific behavioral function-related genes were identified at the single cell level. In another study, Zeisel et al. [114] used large-scale single cell RNA-seq to sequence the neuronal cells from the somatosensory cortex and hippocampal CA1 region, and they identified an interneuron and a postmitotic oligodendrocyte labeled with Pax6 and ltpr2, respectively. Similarly, Tasic et al. [115] defined 49 transcriptomic cortical cell types, including 23 GABAergic, 19 glutamatergic and 7 non-neuronal types based on single cell RNA sequencing. In humans, the structure and function of brain is more complex. Johnson et al. [116] combined FACS and single cell RNA-seq to detect the heterogeneity in evolution of human outer radial glia (ORG). In another study, single cell RNA-seq was used to identify the transcriptome diversity in adult and fetal brains. The results indicated that there was differential gene expression between adult and fetal neurons, and the gradient patterns of gene expression contributed to the understanding of the evolution of neurons in the brain [117]. The latest study used single nucleus RNA-seq to sequence the single neuron from six distinct regions of the human cerebral cortex and identified 16 neuronal subtypes with subtype-specific transcriptome profiles [118]. Additionally, single cell DNA-seq was used for CNV detection in brain diseases. Using single cell DNA-seq, McConnell et al. [119] demonstrated that there were abundant mosaic CNVs in human neurons, especially in hiPSC-derived neurons. In another study, Cai et al. [120] used WGS to find a somatic CNV of chromosome 1q in more than 20% of neurons in a hemimegalencephaly (HMG) patient.

## Conclusions

Biological heterogeneity must be considered in clinical and basic studies. With the advancement of next-generation sequencing, SCS, including single cell genomic, transcriptomic and epigenomic sequencing, has been become the major tool to unlock the secrets of biological diversity [41]. Recently, the application of SCS has been widespread in various research fields, such as cancer, immunology, microbiology, neurobiology and embryogenesis, and many successful commercial kits have emerged in the market [34]. Most exciting is the transformation of the use of SCS from bench to bedside. For example, SCS has been applied to the assessment of human embryos prior to implantation, non-invasive prenatal diagnosis and cancer diagnosis and prognosis [32]. However, there are still several shortcomings of SCS [10, 25]. It is hard to comprehensively and simultaneously sequence the genome, transcriptome and epigenome in a single cell. The high cost of SCS impedes its clinical application, and thus it will be a great challenge for researchers and engineers to provide highly efficient and low-cost technologies in SCS. Furthermore, in situ, real-time and in vivo sequencing and analysis of the DNA and RNA from single cells will be a new field that obtains deep insight into the spatial and temporal measurement of the molecular profiles of single cells. Lastly, new analysis models for the enormous data obtained from SCS should be built to unbiasedly mine the inherent properties of a single cell. Although still evolving, new SCS technologies have become powerful approaches for us to unravel the complexities of nature.
